# Mineralization Potential of Polarized Dental Enamel

**DOI:** 10.1371/journal.pone.0005986

**Published:** 2009-06-19

**Authors:** Reina Tanaka, Yo Shibata, Atsufumi Manabe, Takashi Miyazaki

**Affiliations:** 1 Department of Oral Biomaterials and Technology, Showa University School of Dentistry, Tokyo, Japan; 2 Division of Aesthetic Dentistry, Showa University School of Dentistry, Tokyo, Japan; University of Crete, Greece

## Abstract

**Background:**

Management of human teeth has moved from a surgical to a more conservative approach of inhibiting or preventing lesion progression. Increasing enamel mineralization is crucial in this regard. A potential difficulty is the preferential mineralization of the outermost portion of the enamel that can prevent overall mineralization. We describe a strategy for increasing the mineralization potential of dental enamel.

**Methodology/Principal Findings:**

Extracted human premolar teeth enamel (n = 5) were exposed to a high concentration of hydrogen peroxide with an energizing source. Samples were stored in artificial saliva at 37°C for 1 wk. A desktop X-ray micro-CT system was used to evaluate the mineral density of samples. Mineral distribution was polarized between the lower and the higher mineralized portion of enamel by charged oxygen free radicals due to activation of permeated hydrogen peroxide. The kinetics of energy absorption in the deeper enamel region demonstrated improvement of preferential mineralization into the region without restricting overall mineralization of the enamel. Subsequent increasing mineralization, even in the dense mineralized outer portion of enamel, was also achieved.

**Conclusions/Significance:**

This increased mineralization may promote resistance to acidic deterioration of the structure. The present study is one of the primary steps towards the development of novel application in reparative and restorative dentistry.

## Introduction

Enamel is a hard, nanostructured biocomposite that forms the outer layer of the tooth, offering protection against mechanical damage during dental functions [1]. Continuous balanced demineralization and remineralization are dynamic processes in the enamel of human teeth [2]. If this balance is disrupted, demineralization will progress, leading to a deterioration of the structure through the process known as dental caries [3].

Enamel is the most highly mineralized tissue in vertebrates, and contains 95% to 98% inorganic substances by weight. Hydroxyapatite (Ca_10_(PO_4_)_6_(OH)_2_) in the form of a crystalline lattice is the largest mineral constituent (90–92% by volume). Other minerals and trace elements are present in much smaller amounts. The remaining constituents are organic matter and water [4].

The overall solubility of enamel within the oral environment increases with distance from the enamel surface to the dentin–enamel junction because mineral concentration has been found to be higher near the enamel surface (<100 µm) and decreases dramatically towards the dentin–enamel junction [5]. Mineralization appears to influence the hardness, chemical reactivity, and stability of the enamel while preserving apatite structures [6]. Preferential mineralization of the deeper region of the relatively lower mineralized enamel is therefore important.

Although it is very hard and dense, partial penetration of certain ions and molecules through the hypomineralized enamel structure is possible because it contains small inter-crystalline spaces, rod sheaths, enamel cracks, and other defects [7]. A potential difficulty associated with remineralization of dental enamel is preferential remineralization of the dense mineralized outermost portion of enamel that can prevent complete remineralization by restricting distribution of mineral ions into the deeper region [7], [8], [9].

Energy absorption by enamel heated in a furnace and laser-irradiated enamel has been shown to reduce subsurface dissolution when exposed to acid solutions that simulate the caries process [10]. Explanations of this phenomenon have included chemical modifications to the inorganic and organic components of enamel [11]; and subsequent crystal growth and increase of internal surface area [12]. Energy absorption of the deeper enamel region of vital teeth could therefore be expected to enhance the mineralization potential of enamel.

Hydrogen peroxide has been widely used for cleaning in the papermaking industry and in semiconductor production because of its permeability and environmentally friendly properties [13]. The notable permeability of hydrogen peroxide has even been used to bleach enamel and dentin [14].

Energy absorption of the deeper enamel region would be allowed by activation of permeated hydrogen peroxide using an energizing source because this would increase the rate of decomposition of oxygen to form oxygen free radicals in the region [14].

Quantifying and understanding the mineralization processes and properties of enamel subjected to hydrogen peroxide with an energizing source is the basis of increasing the mineralization potential for enamel. This study aimed to investigate a null hypothesis that energy absorption in the deeper enamel region allows preferential mineralization of the region and subsequent improvement of overall mineralization of enamel. The mineral density of enamel was quantified and visualized with micro-computed tomography (micro-CT) because it provides a non-destructive technique for demonstrating the distribution of minerals in teeth [2].

## Results

**Figure 1:**
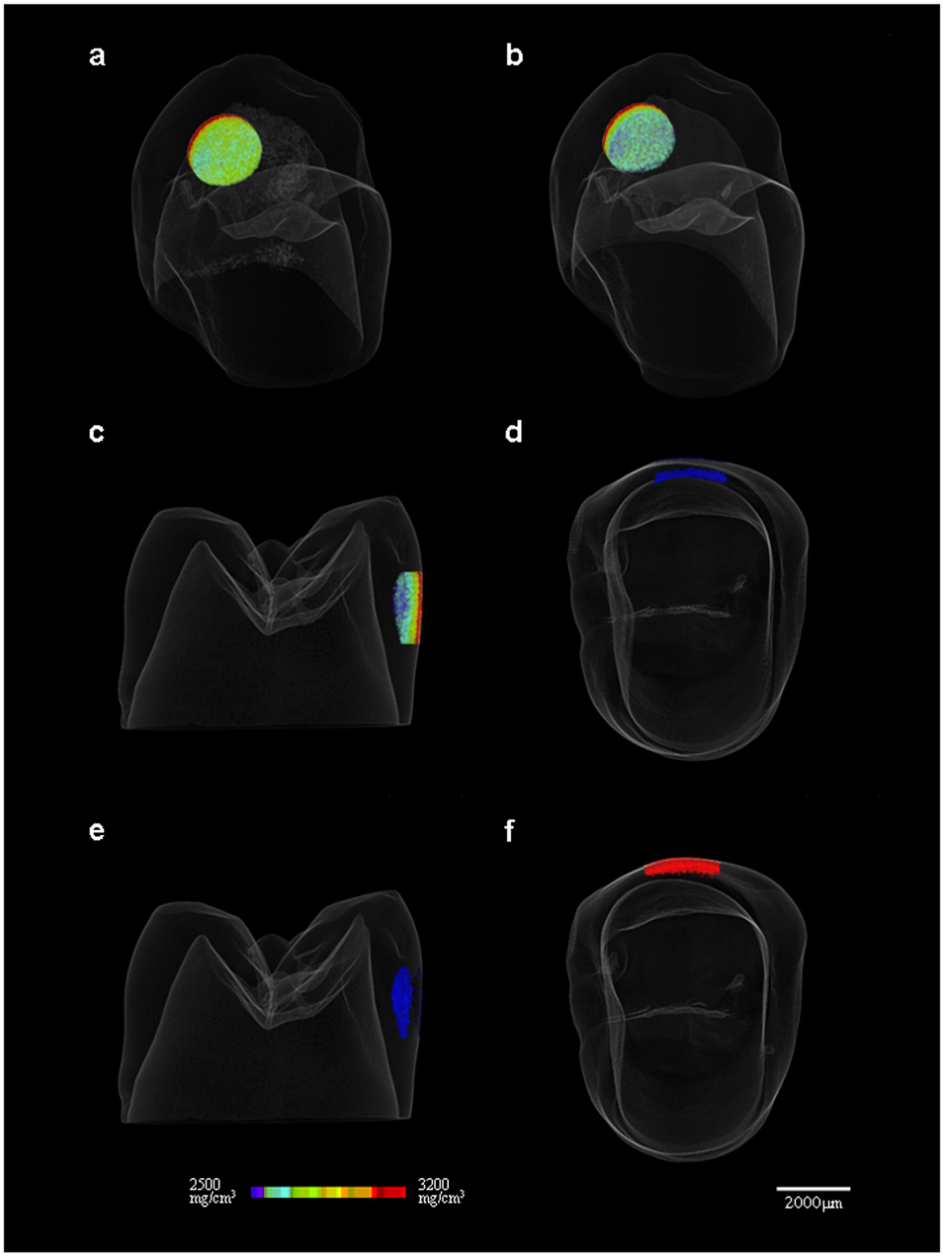
Three-dimensional visualization of the ROI. (a) Color gradation due to the different BMD range 2500–3200 mg/cm^3^ of hydroxyapatite of the untreated sample. (b, c) BMD range 2500–2600 mg/cm^3^ mineral within the ROI of the sample and after exposure to hydrogen peroxide with an energizing source. (d, e) Preferential mineralization of the deeper region (BMD range 2500–2600 mg/cm^3^) of treated enamel was generated after exposure to artificial saliva for 1 d. (f) Enhanced mineralization was seen in the outer portion (BMD range 2900–3200 mg/cm^3^) of the treated sample submerged in artificial saliva for 1 wk; 255-colour gradation of different BMD was assigned according to intensity.

The dense mineralized enamel portion was expressed as a red color, whereas the relatively lower mineralized portion of enamel was blue. A color gradation due to the different BMD (Fig. 1a) of the untreated samples revealed a dense mineralized layer on the outermost portion of enamel whereas the relatively low mineralized portion was towards the deeper region.

**Figure 2:**
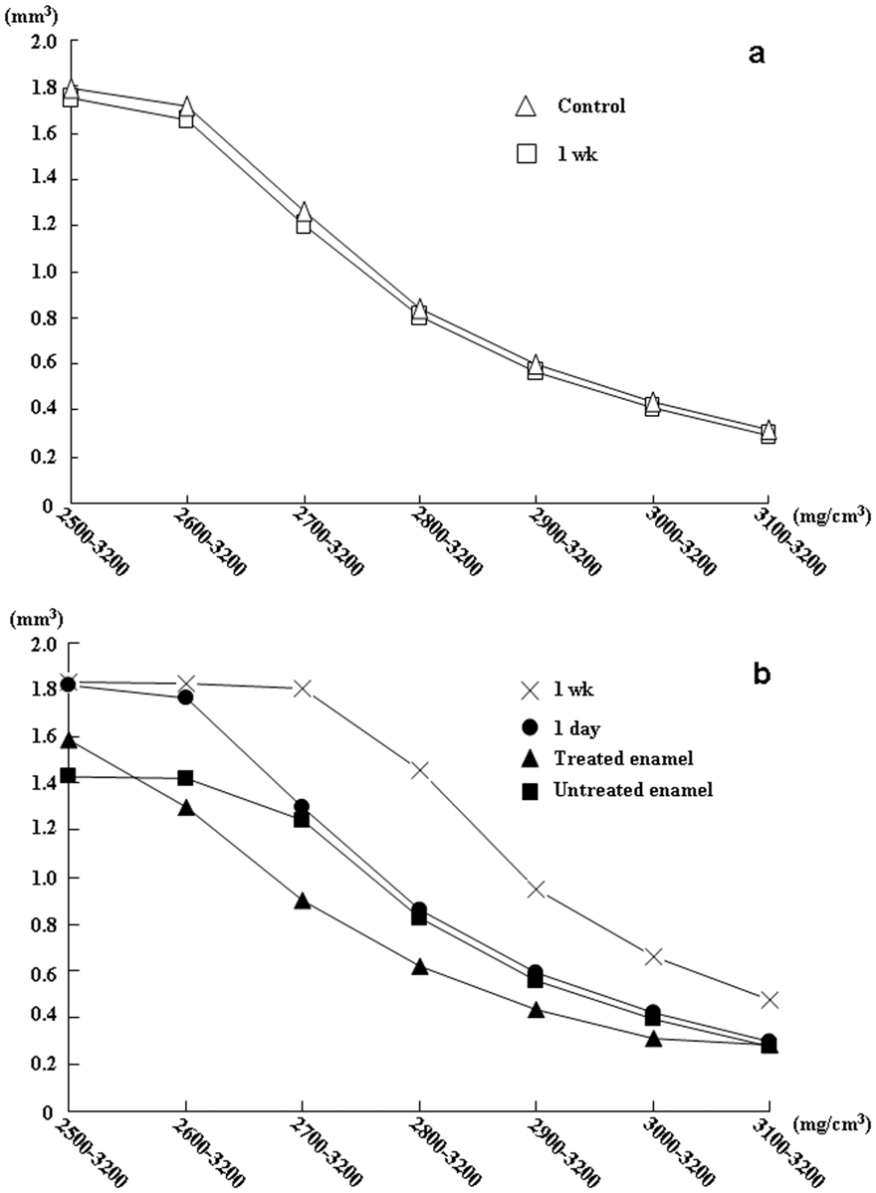
Mineral volume versus BMD range as a function of time. Mineral distribution of BMD range 2500–3200 mg/cm^3^ of hydroxyapatite within the ROI of (a) control and (b) treated sample. Mean values (n = 5) of mineral volume (mm^3^ of hydroxyapatite) between the range 2500 mg/cm^3^ and 3200 mg/cm^3^ of hydroxyapatite (2500–3200 mg/cm^3^ then 2600–3200 mg/cm^3^ up to 3100–3200 mg/cm^3^) within the ROI are shown.

**Figure 3:**
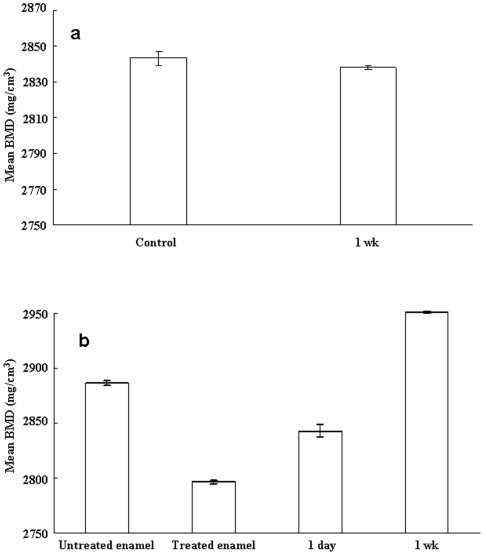
Mean BMD within the ROI. (a) Mean BMD (mg/cm^3^ of hydroxyapatite) within the ROI of control and (b) treated enamel. Results are mean±SD of each result (n = 5), and analyzed by analysis of variance (ANOVA) with a follow-up Tukey test.

The mineral distribution curve (Fig. 2a) and the mean BMD (Fig. 3a) within the ROI of control samples submerged in artificial saliva for 1 wk was not significantly affected (p<0.01) because initial mineralization was achieved before the tests.

The mineral distribution curve of the samples treated with hydrogen peroxide with an energizing source showed larger initial reduction whereas the untreated samples initially showed less reduction (Fig. 2b). The mineral volume within the BMD range 2500–3200 mg/cm^3^ of the ROI of treated samples increased significantly (p<0.01) from 1.43±0.01 mm^3^ to 1.59±0.02 mm^3^ (Fig. 2b), whereas the mean BMD within the ROI decreased significantly (p<0.01) from 2886.1±2.0 mg/cm^3^ to 2796.6±1.5 mg/cm^3^ (Fig. 3b). The mineral distribution curve (Fig. 2b) of treated samples therefore revealed that the mineral volume of the relatively lower mineralized BMD range 2500–2600 mg/cm^3^ increased (p<0.01), whereas it decreased in the range 2600–3200 mg/cm^3^ (p<0.01) except for the dense mineralized portion of samples (3100–3200 mg/cm^3^). A 3D image of the sample revealed that the lower range mineral was dispersed into the deeper region of the ROI (Figs 1b and c).

The mineral distribution curve within the BMD range 2600–3200 mg/cm^3^ of treated samples submerged in artificial saliva for 1 d approached those of untreated samples (Fig. 2b). The mineral volume of treated samples submerged in artificial saliva for 1 d increased significantly (p<0.01) in comparison with untreated samples (Fig. 2b). The mean BMD of the ROI was still lower than that of untreated samples. The mineral volume increased significantly (p<0.01), particularly in the BMD range 2500–2600 mg/cm^3^ within the ROI of samples submerged in artificial saliva for 1 d. The increasing mineral volume within the relatively lower range was seen in the deeper region of the ROI (Figs 1d, e) of samples submerged in artificial saliva for 1 d.

The mean BMD (Fig. 3b) and mineral volume (Fig. 2b) within the ROI of treated samples submerged in artificial saliva for 1 wk eventually reached 2951.1±0.9 mg/cm^3^ and 1.83±0.01 mm^3^, respectively. The mineral volume was not significantly increased (p>0.01) in comparison with treated samples submerged in artificial saliva for 1 d, although the mean BMD within the ROI increased significantly even compared with untreated samples.

Enhanced mineralization was seen in treated samples submerged in artificial saliva for 1 wk. A large mineral distribution was allowed in the range 2700–3200 mg/cm^3^ even in the dense mineralized outermost enamel surface rather than the deeper region of the ROI (Figs 1f, 2b).

## Discussion

In the present study, 3D images and the mineral distribution gradient of untreated samples revealed that a dense mineralized layer was present on the outermost portion of the enamel, and that the mineral density decreased gradually toward the deeper region. The mineral volume versus BMD range shown in Fig. 2 therefore implies mineral distribution towards the dense mineralized enamel surface. This was consistent with a study reporting that inorganic substances vary from the outer enamel surface to the junction between the dentin and enamel [15].

The mineralization of lower mineralized enamel of <2500 mg/cm^3^ in the deeper region of treated samples was improved to >2500 mg/cm^3^ after treatment whereas mineralization in the BMD range 2600–3100 mg/cm^3^ of treated samples was reduced. The most highly mineralized BMD range, 3100–3200 mg/cm^3^, remained even after treatment. A polarization of mineral distribution was therefore generated between the lower and higher mineralized portion.

The content and density of minerals were reported to decrease towards the dentin–enamel junction, whereas enamel protein increased inwards [15]. Hydrogen peroxide is more readily dispersed within the deeper enamel region than in the outer portion due its porosity [14]. This indicates that the polarization associated with energy absorption of the deeper enamel region could be allowed with charged oxygen free radicals by activation of permeated hydrogen peroxide with an energizing source.

Saliva is a natural pH buffer solution that can promote remineralization of enamel in the oral environment. Saliva did not increase mineralization potential because the mineral distribution curve and the mean BMD within the ROI of the control sample submerged in artificial saliva for 1 wk was not significantly affected. The treated sample submerged in artificial saliva expressed considerable positive mineralization even after 1 d.

The mineral volume was significantly increased particularly in the BMD range 2500–2600 mg/cm^3^ of treated samples submerged in artificial saliva for 1 d. The mean BMD of the treated sample for 1 d was still lower than that of untreated samples because mineral volume within the lower BMD range was increased. Preferential mineralization of the deeper region of the treated samples was generated after exposure to artificial saliva for 1 d because the increasing mineral volume within the relatively lower range was seen in the deeper region of the ROI.

The mineral volume of treated samples submerged in artificial saliva for 1 wk was not significantly increased in comparison with treated samples submerged in artificial saliva for 1 d (although the mean BMD within the ROI increased significantly even compared with untreated samples). This revealed that the ROI of treated samples was fully occupied by the mineral within the BMD range 2500–3200 mg/cm^3^, even in treated samples submerged in artificial saliva for 1 d.

The pattern of mineral deposition of treated samples submerged in artificial saliva initially showed that deposition of the lower mineralized region was in the deeper region, and then yielded overall mineral deposition of enamel even in the outermost dense mineralized portion.

The kinetics of energy absorption in the deeper region of polarized enamel allows improvement of the dynamic driving force for mineral deposition to the region. Treated samples submerged in artificial saliva therefore demonstrated preferential mineralization in the deeper enamel region without yielding initial mineralization of the outer portion, which restricts overall mineralization of enamel.

In summary, mineral distribution of enamel is polarized by high-concentration hydrogen peroxide with an energizing source. This situation allows preferential mineralization in the deeper region and subsequent overall enhanced enamel mineralization, which may be effective for preventing incipient caries.

## Materials and Methods

### Specimens

Human premolar teeth extracted for orthodontic indications were obtained under informed consent with a protocol approved by Ethics Committee of Showa University School of Dentistry (Tokyo, Japan).

Samples were stored in a physiological buffered solution. Teeth were then submerged in artificial saliva for 1 d to achieve initial mineralization baseline before the tests. A 4 mm-diameter window centered within the buccal enamel of each tooth was exposed to 35% hydrogen peroxide with appropriate mixed sodium and calcium salt of poly(methyl vinyl ether-maleic anhydride), i.e., poly(MVE/MA). Hydrogen peroxide paste was applied to five samples and activated using a halogen lamp (DP-075, Morita, Tokyo, Japan) for 3 min while the another five samples without treatment were used as control. Treatment was repeated thrice before submerging in artificial saliva [3]. Samples were stored in artificial saliva at 37°C for 1 wk.

### Micro-CT parameters

A desktop X-ray micro-CT system (Shimadzu, SMX-90, Kyoto, Japan) was used to evaluate the mineral density of samples. Samples and five discs of graded hydroxyapatite phantoms were scanned with X-rays generated by a sealed micro-focus X-ray tube (tungsten anode) at 90 KeV and 110 µA with an integration time of 400 s. Samples were rotated over 360° at rotation steps of 0.2°. A 1.0 mm-thick aluminum filter was placed in front of the detector to remove low-energy X-rays.

### Data analysis

The resulting two-dimensional (2D) images (16-bit TIF) were used to reconstruct the digital three-dimensional (3D) object. The data collected were used to reconstruct a 3D image with a resolution of 1024×1024 pixels and with an isotropix voxel size of 25 µm. Appropriate 3D software (TRI/3D-BON, Ratoc System Engineering Company Limited, Tokyo, Japan) was used for visualization and analysis of 3D/volumetric data. The reconstructed data set was imported and displayed for 3D visualization and randomly selected five regions of interest (φ2 mm×500 µm within a 4-mm sample window of enamel) (n = 5). Mean bone mineral density (BMD; in mg/cm^3^ of hydroxyapatite) within the region of interest (ROI) as a function of time was calculated and visualized by the liner relationship between gray level CT values of graded hydroxyapatite phantoms and the ROI. To aid visualization, 255-color gradation of different BMD was assigned according to intensity (Fig. 1).

The mineral volume (mm^3^ of hydroxyapatite) between the range 2500 mg/cm^3^ and 3200 mg/cm^3^ of hydroxyapatite (2500–3200 mg/cm^3^ then 2600–3200 mg/cm^3^ up to 3100–3200 mg/cm^3^) within the ROI was calculated and visualized.

Results were mean±SD of each result (n = 5), and assessed by analysis of variance (ANOVA) with a follow-up Tukey test. p<0.01 was considered significant. These tests were repeated on all samples, and their reproducibility confirmed. Representative sample data are shown.
